# Crafted experiments to evaluate feature selection methods for single-cell RNA-seq data

**DOI:** 10.1093/nargab/lqaf023

**Published:** 2025-03-19

**Authors:** Siyao Liu, David L Corcoran, Susana Garcia-Recio, James S Marron, Charles M Perou

**Affiliations:** Lineberger Comprehensive Cancer Center, University of North Carolina, Chapel Hill, NC 27599, United States; Department of Genetics, School of Medicine, University of North Carolina at Chapel Hill, Chapel Hill, NC 27599, United States; Lineberger Comprehensive Cancer Center, University of North Carolina, Chapel Hill, NC 27599, United States; Department of Genetics, School of Medicine, University of North Carolina at Chapel Hill, Chapel Hill, NC 27599, United States; Lineberger Comprehensive Cancer Center, University of North Carolina, Chapel Hill, NC 27599, United States; Department of Genetics, School of Medicine, University of North Carolina at Chapel Hill, Chapel Hill, NC 27599, United States; Lineberger Comprehensive Cancer Center, University of North Carolina, Chapel Hill, NC 27599, United States; Department of Statistics and Operation Research, University of North Carolina at Chapel Hill, Chapel Hill, NC 27599, United States; Lineberger Comprehensive Cancer Center, University of North Carolina, Chapel Hill, NC 27599, United States; Department of Genetics, School of Medicine, University of North Carolina at Chapel Hill, Chapel Hill, NC 27599, United States; Department of Pathology and Laboratory Medicine, University of North Carolina at Chapel Hill, Chapel Hill, NC 27599, United States

## Abstract

While numerous methods have been developed for analyzing scRNA-seq data, benchmarking various methods remains challenging. There is a lack of ground truth datasets for evaluating novel gene selection and/or clustering methods. We propose the use of *crafted experiments*, a new approach based upon perturbing signals in a real dataset for comparing analysis methods. We demonstrate the effectiveness of crafted experiments for evaluating new univariate distribution-oriented suite of feature selection methods, called GOF. We show GOF selects features that robustly identify crafted features and perform well on real non-crafted data sets. Using varying ways of crafting, we also show the context in which each GOF method performs the best. GOF is implemented as an open-source R package and freely available under GPL-2 license at https://github.com/siyao-liu/GOF. Source code, including all functions for constructing crafted experiments and benchmarking feature selection methods, are publicly available at https://github.com/siyao-liu/CraftedExperiment.

## Introduction

Advances in single-cell RNA sequencing (scRNA-seq) technologies have made a significant impact on biomedical research, leading to the invention and development of many novel single-cell analysis methods. All phases of scRNA-seq data analysis can be affected by these analytical bioinformatic methods, from data preprocessing to final formulation of conclusions based upon observed cell types or cell states. A major challenge in the current field of single-cell analysis is the comparison of alternative analytical methodologies, and in particular, a clear understanding of the contexts in which each method performs the best. Rigorous evaluation of methods in single-cell data is best accomplished using “ground-truth” datasets, where the cell identities are known or derived using robust external information. However, ground-truth datasets are hard to find, and many papers use computationally derived cell labels to evaluate their methods, which can be a circular argument. Another commonly used approach to comparing different methods is simulation. A handful of scRNA-seq data simulation methods have been developed, such as Splatter [1], ZINB-WaVE [2], and scDesign3 [3]. Most of these methods typically start with estimating the parameters from a real dataset and then fit complex statistical models to the data using the estimated parameters and/or additional parameters to generate synthetic datasets. However, as indicated in a benchmark study of scRNA-seq simulation methods [4], many simulation methods failed to maintain a reasonable amount of biological signal. Pure *de novo* simulation of scRNA-seq data tends to be overly artificial because the underlying biology driven by gene expression profiles results in very rich and complex data; this high level of biological complexity is very challenging to effectively simulate.

This motivated us to develop the use of “crafted experiments” for benchmarking various analytical methodologies in scRNA-seq data analysis. The roots of the crafted experiment concept lie in the “semi-experiments” of Hohn *et al.* [5] in the context of internet traffic data analysis. Like scRNA-seq data, the field of internet traffic analysis also involves very complex and hard to simulate data, and therefore careful data manipulation provided many useful insights. In the context of scRNA-seq data, we and others have previously demonstrated the use of crafted experiments in validating newly proposed scRNA-seq methodologies [6,7], but did not formulate a formal definition. Here, we define a “crafted experiment” to be based upon a real dataset, which contains the essential biological complexity. The crafting comes from creating known differences through systematic perturbation, for example of selected gene subsets. A related approach is, real data-based simulations (i.e. adding signal to real datasets), which have been proposed in gene expression analyses (both microarray and RNA-seq studies), in which the majority of their efforts was to create differentially expressed genes (DEGs) between treatment groups to assess differentially expression methods [8–12]. Here, with scRNA-seq data, our goal is to use crafted experiments and study how robust cell types/states are identified. This can be achieved by creating known cell group differences, and then measuring how well each method performs at identifying these groups amongst a sea of real data. An important benefit of crafted experiments is that they allow construction of a full range of test beds with user-defined range of signal-to-noise ratios. Crafted experiments offer a high degree of flexibility because there are many possible ways to do the crafting, for example, perturbing genes, perturbing cells, or creating new patterns, etc.

Feature selection, which is the process of identifying biologically important genes, is a critical step in a scRNA-seq analysis pipeline, as all downstream analyses such as clustering and pseudo-time inference rely on the selected features [13]. Ideally, a good feature selection method should effectively reduce the noise, and more importantly, select informative genes that optimize the separation between biologically distinct cell types. However, feature selection has been paid relatively little attention and there is a lack of systematic comparison of various methods. The most popular method called highly variable genes (HVGs) has been widely used and recommended as the “best practice” by Luecken and Theis [14]. There are different variants of the HVG approach, but all are based on the idea of modeling the relationship of mean and variance and selecting HVGs whose variance exceeds a null model derived from the data. For example, Seurat V2 [15] bins the genes based on their average expression, and calculates Z-scores for the *gene dispersion* (defined as variance divided by the mean, sometimes called the coefficient of variation) within each bin. The HVGs are then identified based on the Z-scores within each bin, and ultimately some are selected from each bin. Seurat V3 [16] applies a “Variance Stabilizing Transformation (VST)” to provide the correct mean-variance relationship. Alternatively, approaches that are based on the *proportion of zeros* (i.e. dropout rate) have been developed, such as M3Drop [17] and HIPPO [18], with the goal of finding genes that have more zeros than expected. M3Drop models the relationship between the gene mean and fraction of zeros using a Michaelis–Menten function, whereas HIPPO models the UMI counts as a mixture of Poisson distributions, and uses a zero-inflation test to select genes by their deviation from the Poisson model. In a similar deviance spirit, Townes *et al.* [19] proposed to select features according to their deviance from a multinomial model.

To date, only a few systematic benchmarking studies have been conducted [20, 21]. For example, Yip *et al.* [20] compared seven HVG methods from six software packages and revealed high discrepancies between methods in downstream clustering performance. Thus, ongoing efforts are needed to improve feature selection methods in scRNA-seq data analysis, as well as to systematically assess the impact of different feature selection methods on the downstream analyses.

Here we present a novel feature selection framework for clustering scRNA-seq data using goodness-of-fit measures (GOF). The main idea is to select features based on the GOF of raw UMI count data to a mixture of negative binomial (NB) distributions, termed “average negative binomial” (ANB). The ANB distribution models generic variation such as cell library effects, so departures from ANB indicate important further structure such as cell types. We develop four variants of GOF in terms of how the GOF is quantified. We use the crafted experiments to compare different variants of GOF as well as GOF against other state-of-the-art feature selection methods.

## Materials and methods

### Datasets and preprocessing

To benchmark our newly developed feature selection method, we used six real scRNA-seq datasets that can be classified into two groups: datasets with external cell labels (three-cell line mixture dataset, Zheng4eq, Zheng4uneq, and Zheng8eq datasets) and datasets with computationally derived cell labels (FVB3 mouse mammary gland dataset and Zilionis human lung immune dataset). The three-cell line mixture (P3CL) dataset generated in our lab [22]; for this dataset, we have two pieces of information providing ground truth cell identities: (1) the mixing ratio of the cell lines (1:3:6) and (2) SNPs discovered from bulk RNA-seq data unique to each cell line. The set of Peripheral Blood Mononuclear Cells (PBMC) datasets were obtained from Zheng *et al.* [23], where homogeneous PBMC populations were purified using fluorescence-activated cell sorting (FACS), therefore the true cell identifies can be obtained externally based on the FACS purifications. The Zheng4eq dataset contains 3994 cells sampled from four purified cell populations (B cells, CD14 monocytes, naïve cytotoxic T cells, and regulatory T cells) in equal proportions. The Zheng4uneq dataset consists of the same four cell types, but in different proportions, with B cells, CD14 monotypes, naïve cytotoxic T cells and regulatory T cells at a 2:5:1:2 ratio. The Zheng8eq dataset consists of 3994 cells sampled from eight purified cell populations (B cells, CD14 monocytes, CD4 T helper, CD56 Natural killer cells, memory T cells, naïve cytotoxic T cells, naïve T cells, and regulatory T cells) in equal proportions.

The FVB3 mouse mammary gland dataset contains 8 459 cells. The Zilionis human lung immune dataset [24] contains 15 939 cells and to speed up the runtime, we randomly selected 5000 immune cells. These were classified as B cells, Mast cells, MoMacDC cells, Neutrophils, NK cells, pDC, Plasma cells, RBC, and T cells according to the computationally derived cell labels.

For all of the datasets, genes with zero counts in all cells were discarded. Preprocessing for the three-cell line mixture dataset and the FVB3 mouse mammary gland dataset follows the preprocessing steps described in the MultiK paper [6]. In particular, cells were filtered out if any of the number of total counts per cell, the number of detected genes, or the proportion of expressed mitochondrial genes was away from their medians by more than ± 3 times the median absolute deviation. No cell filtering was performed on the other datasets because they were public datasets and already previously filtered by the authors. For all datasets, we applied the Seurat (RRID:SCR_016341) best practices pipeline prior to feature selection using 30 PCs.

### Benchmarking of feature selection methods

All feature selection methods were implemented following their guided tutorials. Default parameters were used everywhere. Because the number of selected features can impact the cluster analysis, we selected the top 2000 features according to the criteria used to rank the genes for each method except for HIPPO. HIPPO uses a cutoff threshold in the zero proportion test, and therefore, there may not be 2000 genes selected by HIPPO in some datasets.

To evaluate the performance of each feature set in the crafted experiments, we first applied MultiK using the selected features from each method using all cell groups and evaluated each method only based on the estimated optimal number of groups in the low-resolution space. We work here with the low-resolution MultiK results because that is where the crafting was performed. To rank each method, we compared the MultiK low-resolution space results to the expected number of groups according to the external cell labels. The method whose low K solution is the closet to the expected group number gets a lower rank. If methods have the same performance, we assign a tied rank. In addition to applying MultiK, we compared methods using DiProPerm Z scores to assess the statistical strength of the difference between the crafted and the original luminal group only. The mean difference version of DiProPerm was used with 100 permutations. Methods with higher Z scores get lower ranks.

## Results

### Construction of crafted experiments

**Figure 1.**
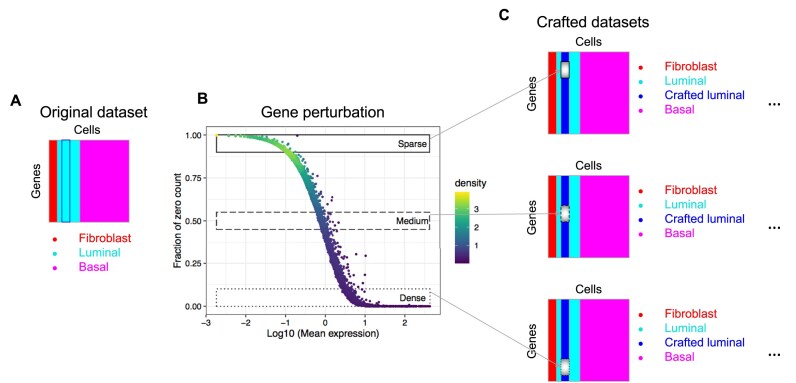
Illustration of crafted experiments. (**A**) A crafted experiment is based upon a real dataset with known cell labels (Fibroblast, Luminal, and Basal). (**B**) Gene perturbation strategies. To create a crafted group, the crafted genes can be selected from different gene regions (represented in boxes) based on the relationship between the average gene expression across cells and the proportion of zeros per gene across cells. Each dot is a gene, and the color of the dot represents the two-dimensional kernel density estimate. Sparse region is defined as the proportion of zeros per gene across the crafted cells between 0.9 and 1; Medium region is defined as the proportion of zeros per gene across the crafted cells between 0.45 and 0.55; Dense region is defined as the proportion of zeros per gene across the crafted cells between 0 and 0.1. (**C**) A deliberate cell group (Crafted luminal) is created by perturbing a set of genes (small box) for a set of cells.

The basic concept of how to construct a crafted experiment is illustrated in Fig. 1, where we start with a real dataset with ground truth cell labels (Fig. 1A). In our example dataset, there are three cell types; this represents the non-crafted portion of this data set that was created by purposely mixing together three distinct human cell lines (i.e. MCF7, SUM149 and dermal fibroblasts [22], thus providing robust external known labels). Then, we “spiked in” signal into this existing dataset by perturbing a set of genes (indicated by small rectangles in Fig. 1C) for a subset of the luminal cells so that a “known difference” is created (Fig. 1C). The deliberate group of cells (crafted luminal) can and is crafted in many ways. One approach is random selection of gene sets. However, this gave less useful insights in our experiments because more than 50% of genes have their proportion of zeros across cells greater than 0.9 (i.e. more than 90% of cells have a count of zero per gene), resulting in manipulation of mostly genes with many zeros (i.e. the sparse genes). A more useful approach to choose crafted gene sets is illustrated in Fig. 1B, where the relationship between the average gene expression across cells and the proportion of zeros per gene across cells is plotted. In our experiments, we focus on the genes in the boxes in Fig. 1B. This resulted in gene sets with quite different but very relevant characteristics. This focusing on genes from different sparsity regions gives more control over the type of genes that are being crafted, resulting in more insightful analytical validations. It also allows us to study how typically relevant genes with high and medium expression levels across cells can affect crafting of the new cell group.

**Figure 2.**
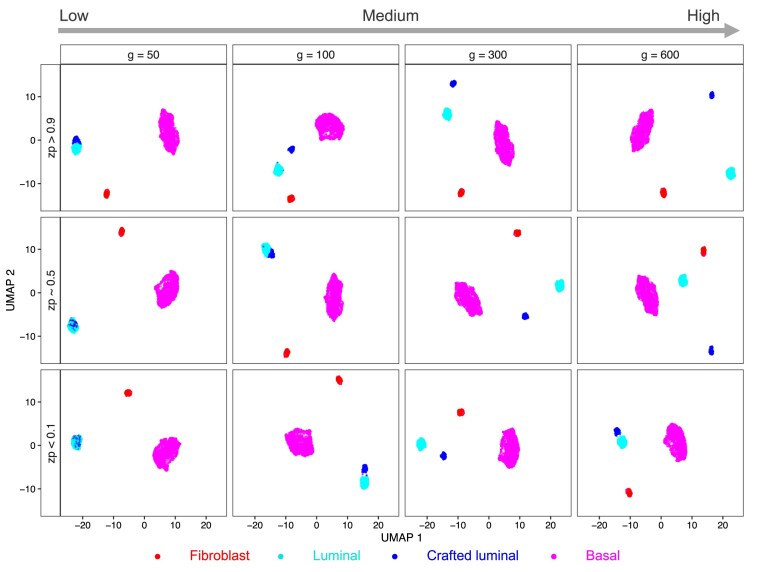
UMAP plots showing the crafted artificial cluster in representative crafted experiments. Each dot is a cell and the colors of the dots indicate the cell's identity. Columns represent the number of perturbed genes in each crafted experiment. Rows represent the region of genes being perturbed and its corresponding gene perturbation. In the first row, the genes are perturbed by adding counts generated from Poisson (0.5) to the original counts. In the second row, the genes are perturbed by adding count of 2 to the original counts. In the last row, the genes are perturbed by adding counts generated from Poisson (count*1.5) to the original counts. zp = zero proportion per gene.

Specifically, for the crafted experiments generated in this paper, we used the three-cell line mixed data set (Fibroblast, Luminal, and Basal cell lines mixed at 1:3:6 ratio) from Dong *et al.* [22]. Because the luminal cells tended to be homogeneous [6] and there is a reasonable number of them, we chose to craft a randomly sampled 30% of the luminal cells (*N* = 162) and modified a set of genes for these cells in various ways as to create a “crafted luminal” group. We focused on crafting genes from three major regions: (1) Sparse (S)—genes with proportion of zeros greater than 0.9 (*N* = 9 223); (2) Medium (M)—genes with proportion of zeros between 0.45 and 0.55 (*N* = 732); (3) Dense (D)—genes with proportion of zeros less than 0.1 (*N* = 613). From each gene region, we randomly selected 600, 300, 100, and 50 genes, and for best comparisons, these sets of genes were all nested within each other. To create the new crafted cell group, we applied three types of perturbation to the selected genes in the crafted cells: adding a fixed number of counts to the original counts (${c_{ij}}$), adding random Poisson(λ) counts to ${c_{ij}}$ for various values of λ, and adding random Poisson($f*{c_{ij}}$) counts to ${c_{ij}}$ for various values of $f$. Further details are in Methods – Crafted experiments section. As detailed in Table 1, we generated 24 crafted datasets and the UMAP (Uniform Manifold Approximation and Projection) visualization of 12 of them is shown in Fig. 2, which used standard HVG feature selection and Seurat UMAP projections. In general, from left to right in each row, there is more separation of the crafted luminal group from the original luminal group; in general, as the number of genes being perturbed increases, the underlying strength of the crafted cluster increases.

**Table 1.** A list of crafted experiments generated from this paper based on the three-cell line mixture dataset

Gene region	Number of perturbed genes	Type of perturbation and parameter
Sparse (zp > 0.9)	50, 100, 300, 600	Adding counts generated from Poisson (0.5)
Medium (zp ∼ 0.5)	50, 100, 300, 600	Adding count of 2
Medium (zp ∼ 0.5)	50, 100, 300, 600	Adding counts generated from Poisson (1.5)
Medium (zp ∼ 0.5)	50, 100, 300, 600	Adding counts generated from Poisson (3*count)
Dense (zp < 0.1)	50, 100, 300, 600	Adding count of 10
Dense (zp < 0.1)	50, 100, 300, 600	Adding counts generated from Poisson (1.5*count)

zp = proportion of zeros per gene across cells.

### Overview of GOF workflow

The main motivation for developing the GOF framework to select informative features for robust identification of cell types/subtypes is that scRNA-seq data are highly sparse with more than 80% of the data matrix entries being zeros. Moreover, the data are quite complex due to cell-to-cell heterogeneity as well as technical noise. Therefore, it is critical to develop statistical methods for accurately modeling scRNA-seq data. Several recent studies have shown that the counts in the individual entries of the scRNA-seq data matrix can be well modeled as independent Poisson random variables, with each entry having its own Poisson parameter [18,19,25]. Thus, summing these counts over each gene has a distribution that is a mixture of Poissons. For genes that tend to have similar effects across cells, this mixture behavior is mostly driven by multiplicative factors, such as sequencing library effects. To model these common multiplicative effects across genes, we propose to fit an ANB distribution as detailed in the Methods (see GOF methodology section).

**Figure 3.**
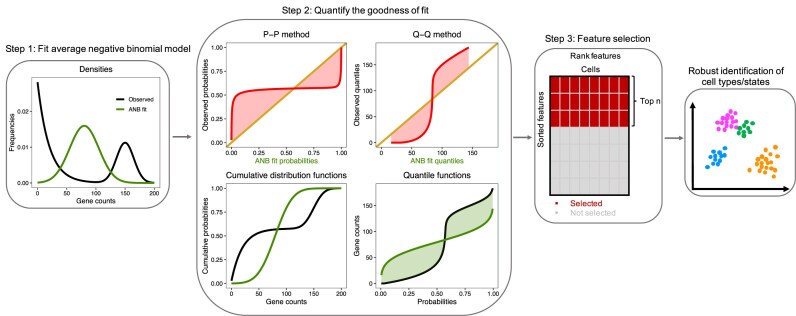
Overview of GOF framework. Feature selection is performed in three steps. In step 1, GOF fits an ANB distribution to the observed gene count distribution for each gene. In step 2, GOF uses multiple approaches to quantify the GOF: P-P and Q-Q methods (top), and 1-Wasserstein distance metric (bottom). In the P-P and Q-Q plots, the 45-degree line indicates the fitted distribution and the observed distribution are identical. The curves are the CDF of the ANB distribution against the CDF of the observed distribution (in the P-P plot), and the quantile of the ANB distribution against the quantile of observed distribution (in the Q-Q plot). GOF then computes the Area Between the Curves (ABC) and uses it as the criterion to rank the features. The 1-Wassertein distance is thought of as the cost of moving probability mass shown in the CDFs (bottom left) and summarized by the L-1 norm between the corresponding quantile curves shown in the bottom right. A gene with a large ABC value or a gene with a large 1-Wasserstein distance indicates a strong departure from the ANB distribution due to systematic biological variation, and this type of gene is selected for the downstream clustering analysis (step 3).

An overview of the GOF workflow is shown in Fig. 3. In step 1, GOF first fits an ANB distribution to the observed count distribution for each gene. In step 2, GOF assess the quality of the mixture fit using various approaches. GOF starts with visual techniques, the Probability-Probability (P-P) plot and the Quantile-Quantile (Q-Q) plot. The P-P plot is a variation of the familiar receiver operating characteristic (ROC) curve. Specifically, the P-P plot is constructed as plotting the cumulative distribution functions (CDF) of the ANB fitted distribution against the CDF of the observed count distribution (the curve in the P-P plot). The 45-degree line indicates the two distributions are identical. The Q-Q plot is the corresponding quantile version of the P-P plot. Both P-P and Q-Q plots indicate quality of fit in terms of their closeness to the diagonal 45-degree line. For both of these distributional assessments, the overall quality is summarized by the area between curves (ABC), which is the criterion for ranking genes. GOF then ranks the features according to the ABC values. A gene with a large ABC value indicates a strong departure from the ANB distribution due to systematic biological variation.

In addition to the P-P and Q-Q approaches, we also propose computing the 1-Wasserstein distance to compare the observed gene count distribution to the expected ANB distribution. The 1-Wasserstein metric arises from the idea of optimal transport [26, 27], and it is often thought of in terms of the cost of moving probability mass from one distribution to the other. Intuition is illustrated in the bottom row in Step 2 of Fig. 3. Horizontal movement of probability mass is shown in the CDF in the left panel. This corresponds to vertical movement in the corresponding quantile curves shown in the right panel, which is summarized by the L-1 norm to give the 1-Wasserstein metric. Here, both the observed and the fitted distributions are discrete and supported on the non-negative integers, so computing the 1-Wasserstein metric between discrete probability distributions simply involves sums. Due to scaling, we further adjust the 1-Wasserstein distance metric by gene average or gene median. Further details are provided in Materials and methods—GOF methodology section. In step 3, GOF ranks features based on the GOF measures computed in step 2, namely the GOF ranks the genes based on the ABC values from the P-P or Q-Q method and on the 1-Wasserstein distance metric. Then GOF selects the features with the highest values for downstream clustering analysis. In this paper, we use the top 2000 features as commonly used in the field.

### Crafted experiment applications

An important use of crafted experiments is comparison of our newly developed feature selection methods with each other and with more standard methods. Here, we benchmark our feature selection methods against 4 previously established methods: Seurat.vst [16], Seurat.disp [15], HIPPO [18], and the Townes *et al.* deviance-based method (called devianceFS here) [19] on their ability to identify biologically relevant features, and the crafted group, using cluster analysis. Within our GOF method, we also compare four variants: P-P method (PP.ANB), Q-Q method (QQ.ANB), 1-Wasserstein metric adjusted by gene mean (Wdist.mn), and 1-Wasserstein metric adjusted by gene median (Wdist.med). A list of benchmarked methods can be found in Supplementary Table S1.

**Figure 4.**
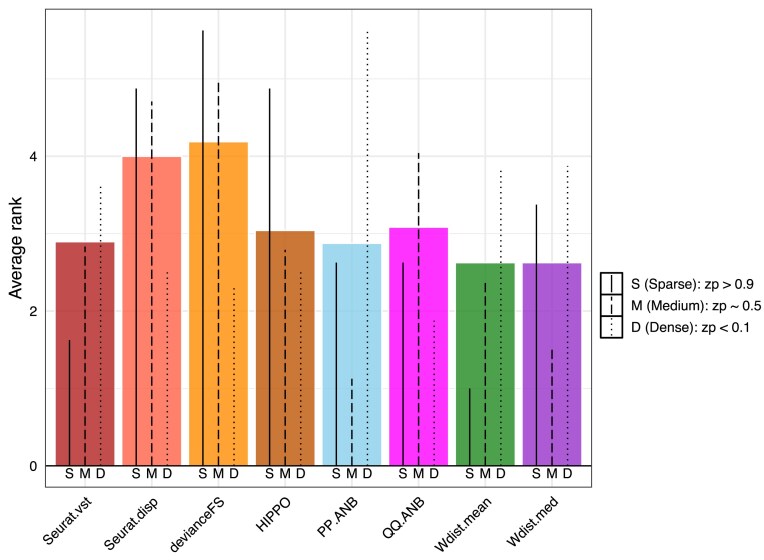
Evaluation of feature selection methods for finding features that identify the crafted artificial cluster in crafted experiments. Feature selection methods are ranked by averaging their overall performance across crafted experiments on their ability to separate the crafted artificial cluster. Two evaluation metrics are used to quantify the performance (MultiK and DiProPerm Z-scores). Each colored bar represents a feature selection method. The heights of the colored bars represent overall ranks with lower rank indicating better performance. The lines within each bar indicate ranks within a method in different perturbed gene regions.

We applied each feature selection method to the 24 crafted datasets and evaluated their performance in terms of their ability to find each newly crafted cluster. To quantitively assess how well each feature set can identify the cell groups, we used two evaluation methods: MultiK [6] and DiProPerm [28]. MultiK is a method previously developed by our group that determines the optimal number of clusters using a multi-resolution perspective. We applied MultiK using the features selected from each feature selection method to see how well the selected genes can identify the correct number of cell clusters. Direction-Projection-Permutation (DiProPerm) is a hypothesis test for comparing two high-dimensional distributions. We applied the DiProPerm to test if there was a statistically significant difference between the crafted luminal group and the original luminal group based on the genes identified by each feature selection method. We then assigned a ranking to each feature selection method based on their performance in each specific crafted experiment. To summarize the overall performance, we computed the average rank for each feature selection method across all 24 crafted experiments and evaluation metrics (colored bars in Fig. 4) where a lower rank indicates a better performance. Compared to the field-standard methods Seurat.vst (dark red bar), three of our GOF variants (Wdist.mean, PP.ANB, and Wdist.med) had better overall performance. Because these crafted experiments were constructed using genes selected from different regions (Sparse, Medium, and Dense in terms of proportion of zeros per gene across cells), we further studied the performance of each method in crafted experiments constructed from each gene region (black lines within each colored bar). In the sparse region, Wdist.mean (solid line in the green bar) performed the best followed by Seurat.vst (solid line the dark red bar); however, in the dense region, QQ.ANB (dotted line in the pink bar) had the best performance, followed by devianceFS (dotted line in the orange bar). This is expected because it has been reported that the devianceFS method tends to select highly expressed genes [19]. In the medium sparsity region, our PP.ANB method performed the best, with our two Wdist methods being second and third. Altogether, both Wdist.mean and Wdist.med methods performed the best across the entire range of signals in the crafted experiments. The PP.ANB method performed the best when perturbing genes with medium zero proportion, and the QQ.ANB performed the best when perturbing genes with high total counts. Overall, our Wdist.mean method (green bar) and Wdist.med (purple bar) had the best performance and devianceFS (orange bar) had the worst performance.

In addition to the evaluation metrics used above, we employed another orthogonal approach to assess the feature selection performance. Given that specific gene sets were perturbed to establish a ground truth group, we calculated the number of crafted genes selected and not selected by each feature selection method for each crafted dataset as well as the number of uncrafted genes selected and not selected (Supplementary Files 1-6). We plotted the proportion of crafted genes selected by each method for each crafted dataset in Supplementary Fig. S1. Consistent with the results shown above in Fig. 4, we observed that the devianceFS, and QQ.ANB methods selected a higher proportion of crafted genes in the dense region, indicating better performance of these methods. In the Sparse region, Widst.mean selected the highest proportion of crafted genes, suggesting best performance across all methods. However, in the medium sparsity region, PP.ANB did not select the highest proportion of crafted genes but Wdist.med and Seurat.vst had an overall higher proportion of crafted genes.

### Benchmarking using experimental datasets

**Figure 5.**
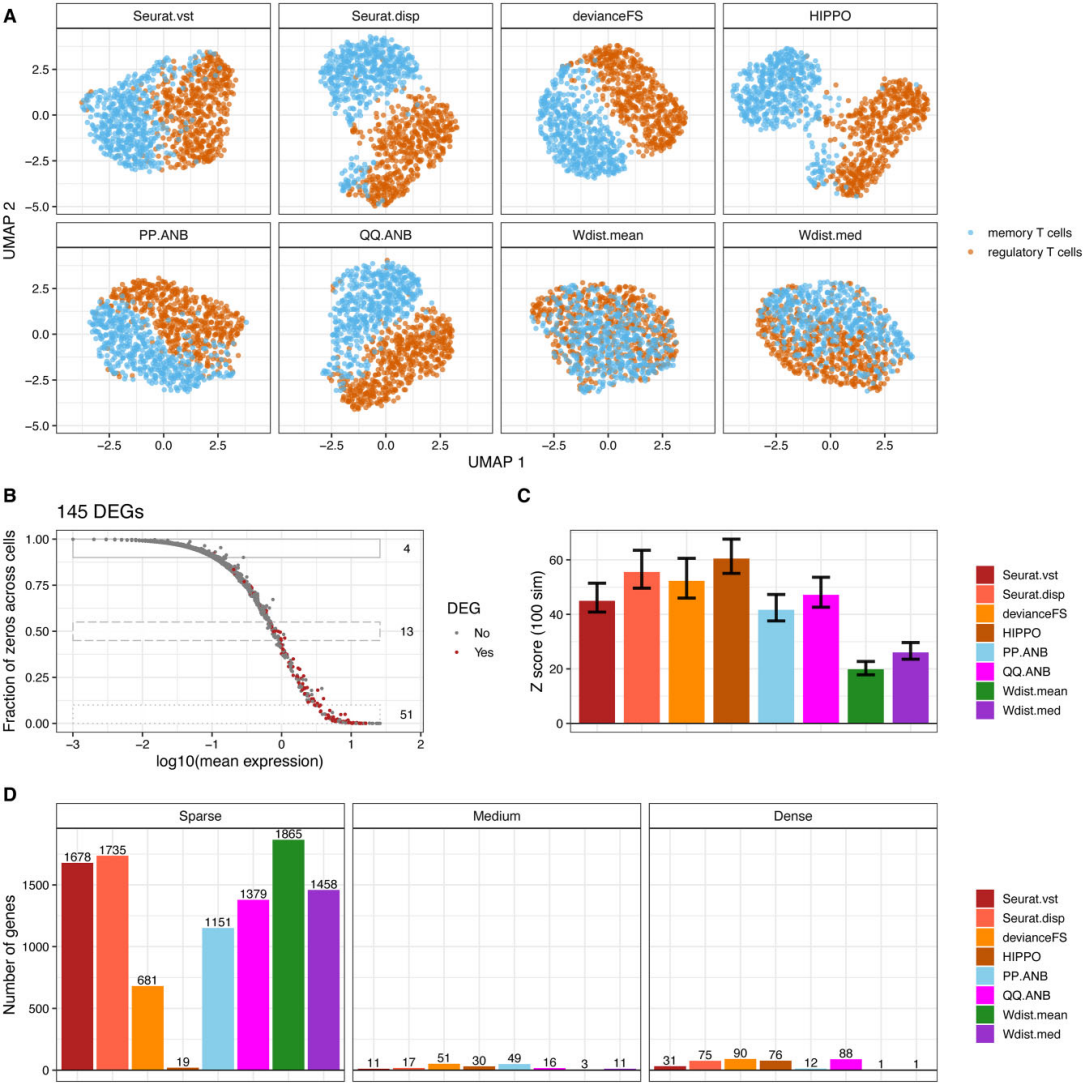
Benchmarking feature selection methods in Zheng8eq T cell pairs. (**A**) UMAP of memory T cells and regulatory T cells using the top 2000 features selected from different feature selection methods in the Zheng8eq dataset. (**B**) Scatterplot of gene mean expression across cells on log10 scale and proportion of zeros per gene across cells. Each dot is gene, and DEGs between the memory T cells and regulatory T cells are highlighted in red. A total of 145 DEGs were identified using Wilcoxon test implemented in the FindMarkers() function from R/Seurat package. Boxes indicate the sparse, medium, and dense region. The numbers on the right side of each box indicate the numbers of DEGs in each region. (**C**) DiProPerm Z scores and their 95% confidence intervals for each feature selection method from 100 simulations. The mean difference direction between the two groups was computed from the first 30 principal components which were calculated from using the top 2000 selected features from each method. (**D**) Barplot of the number of genes in the top 2000 genes selected from each feature selection method in sparse, medium, and dense region.

To assess the clustering performance of these various feature selection methods in real non-crafted experimental contexts, we benchmarked each method on six published datasets. These datasets include the three-cell line mixture (P3CL) dataset which was used in our lab for the crafted experiment [22] as well as Zheng4eq [23], Zheng4uneq [23], Zheng8eq [23], FVB3 [22], and Zilionis human [24]. Three of the new datasets have external cell labels (Supplementary Figs S2–S4), and two have computationally derived cell labels. We applied MultiK [6] to assess the ability of each method to find the groups in the data (Table 2). MultiK estimates optimal numbers of clusters in the data, and it usually provides two optimal k values (one in low-resolution space and one in high-resolution space), but sometimes it can give up to three optimal k values. In general, in the datasets with external cell labels, all methods performed similarly in the low-resolution space and most methods were able to find the expected number of groups. Specifically, in the three-cell line mixture dataset, all methods performed well in the low-resolution space; in the high-resolution space, Seurat.vst and devianceFS tended to call more groups than the other methods. In the Zheng4eq dataset, devianceFS, HIPPO and Wdist.med identified more than 4 groups in the low-resolution space. In the Zheng4uneq dataset, HIPPO, PP.ANB, Wdist.mean and Wdist.med identified more than 4 groups in the low-resolution space. However, in the Zheng8eq dataset, none of the methods identified 8 groups in the low-resolution space. We think this is not totally unexpected as some of the groups in this dataset are quite distinct from each other such as B cells, CD14 monocytes and CD56 natural killer cells, and some are much more similar such as CD4 T helper, memory T cells, and regulatory T cell (Supplementary Fig. S4). To further quantify how well each method can separate various groups in this dataset, we performed DiProPerm analysis on different pairs from this dataset. We observed that for the pair B cells vs. regulatory T cells, their difference was mainly driven by genes in the Sparse region. In particular, 261 out of 600 DEGs were in the Sparse region in Supplementary Fig. S5A and B. The Seurat.disp and HIPPO methods had the highest DiProPerm Z scores, followed by the Wdist.mean and Seurat.vst methods (shown in Supplementary Fig. S5C). That was consistent with the benchmarking results in the crafted experiments (Fig. 4), where we found that the Wdist.mean and Seurat.vst methods performed well in the crafted experiments where genes in the sparse region were perturbed. For the pair memory T cells versus regulatory T cells, the difference was mainly driven by genes in the Dense region, with 51 out of 145 DEGs in the dense region in Fig. 5A and B. The Seurat.disp and HIPPO methods showed the highest DiProPerm Z scores, followed by the devianceFS and QQ.ANB methods (shown in Fig. 5C). This was consistent with the good performance of the QQ.ANB and devianceFS methods in the crafted experiments where genes in the dense region were crafted (Fig. 4). Furthermore, as shown in Fig. 5D and Supplementary Fig. S5D, the Wdist.mean method tended to select genes in the sparse region whereas the QQ.ANB and devianceFS methods tended to select genes in the dense region.

**Table 2.** Benchmarking of feature selection methods using MultiK

	Datasets with external labels				Datasets with computational labels	
	P3CL (*k* = 3)	Zheng4eq (*k* = 4)	Zheng4uneq (*k* = 4)	Zheng8eq (*k* = 8)	FVB3	Zilionis human
Seurat.vst	3, 11	4, 6, 11	4, 5, 10	5, 9	8, 18	7, 22
Seurat.disp	3, 7	4, 5, 7	4, 5, 10	4, 6, 10	9, 16	8
devianceFS	3, 11	5, 6, 11	4, 5, 13	4, 8	10, 24	7, 8
HIPPO	3, 7	5, 12	5, 15	5, 7, 9	9, 15, 19	6, 14
PP.ANB	3, 6	4, 5, 12	5, 11	4, 5,10	9, 17	8, 15
QQ.ANB	3, 7	4, 5, 10	4, 5, 10	4, 9	10, 17	8, 20
Wdist.mean	3, 7	4, 5, 10	5, 10	5, 6	8, 18	7, 22
Wdist.med	3, 7	6, 11, 12	5, 9, 11	4, 5, 10	9, 10, 16	8, 15

*k* under the name of each dataset indicates the number of clusters.

In the datasets where only the computationally derived cell labels are available, due to the lack of ground truth, we compared the performance of each method to Seurat.vst (the field standard method). We found that the Wdist.mean method performed most similarly to the Seurat.vst method, with the PP.ANB and QQ.ANB methods somewhat similar (Table 2). We also found that devianceFS behaved very differently from all the other methods. The reason for this behavior is likely due to the sparsity of the datasets, as we observed that the FVB3 (mouse mammary gland) dataset has the lowest sparsity among the datasets we considered, and the devianceFS method tends to find more highly expressed genes, so it was not surprising that more cell groups were found by this method (Supplementary Fig. S6, Table 2). In the Zilionis human lung dataset [24], most methods performed similarly to Seurat.vst in the low-resolution space; however, in the high-resolution space, devianceFS, HIPPO, PP.ANB and Wdist.med identified far fewer groups than Seurat.vst.

## Discussion

Comparison of data analytical methods remains a key challenge in single-cell analysis. This is mainly because ground truth datasets (datasets with robust external cell labels) are hard to obtain and simulating scRNA-seq data is very difficult due to the richness and complexity of both the underlying biology and technical factors in the data. We propose the use of “crafted experiments” for benchmarking different scRNA-seq methods. A crafted experiment is based upon a real dataset but is augmented by the experimenter through adding in some signals to create a “known difference” between the biologically distinct groups. The use of crafted experiments offers numerous advantages. First, crafted experiments allow comparison of methods without relying on computationally derived cell labels, which are of course influenced by the properties of a given gene selection and clustering methods. Second, crafted experiments preserve the complexity of the real data and enable experimenters to apply a range of perturbations from weak to strong in a known way to control the signal versus noise ratio. Lastly, crafted experiments allow the understanding of contexts in which a certain method outperforms the others and vice versa.

Feature selection plays a key role in the downstream scRNA-seq analysis such as clustering but is often paid less attention to. Most existing methods are based on the idea of controlling for the relationship between gene mean and variance and select features with the largest variance and calling them HVG. However, this approach is sensitive to normalization as raw counts are first normalized before applying the HVG procedure. Thus, novel feature selection methods are needed. Here, we develop a new feature selection framework called GOF that models scRNA-seq data using an ANB distribution and selects features based on GOF measures. GOF has several novelties. First, we developed a new distribution termed ANB for modeling gene count distributions in scRNA-seq data. The advantage of fitting an ANB distribution over the NB distribution is that the ANB model reduces noise in parameter estimation by taking into account the special structure of the data and using a pooled parameter estimate. In contrast, NB model estimates individual parameters for each gene separately, which tends to result in overfitting of the data, especially for the low count genes that are expressed in only a small subset of cells and are modeled with high variance [29]. Second, to our best knowledge, GOF is the first method that exploits visual assessment of model fit via P-P and Q-Q plots and 1-Wasserstein metric for feature selection. These visually oriented methods tend to have more statistical power in the important directions that are visually clear, as compared to methods that are simply based on ranking summary statistics calculated from the empirical distribution. The 1-Wasserstein metric appears to be more powerful and robust in finding genes across a wide spectrum of crafting. Lastly, GOF provides a general framework for feature selection in scRNA-seq data. For the feature selection purpose, others may build upon this framework by adding other distribution types for modeling scRNA-seq data or extend this framework to other types of single-cell data such as single cell protein, chromatin, or spatial data. In addition, the proposed ANB model can also be used for other applications such as clustering. For example, one can replace the original data matrix with the ANB fit data and apply clustering to identify biologically meaningful cell types or states.

Despite the advantages of GOF, we noted some limitations. First, as is common in many other methods, GOF still relies on the user's choice of several parameters. These include the number of genes to select that was arbitrarily set at 2000 in our examples here. It is not clear how this user-subjective choice impacts further cluster analysis. Second, as shown in the results from both the crafted experiments and the real experimental datasets, among all the GOF variants, each method performs the best in certain contexts. It is possible that an approach that effectively combines all these variants may lead to improved performance.

In conclusion, we demonstrate that the use of crafted experiments for benchmarking our newly developed feature selection methods against standard methods, which provides a robust computational framework for assessing various analytical methods and identifies in which contexts a certain method performs better than others and vice versa. We show that our newly developed Wdist.mean method has the best overall performance in identifying cell groups in the crafted experiments across a range of “spiked-in” signals. Our PP.ANB performs the best in the crafted experiments where the genes with medium proportion of zeros are being perturbed, and our QQ.ANB has the advantage of finding the crafted group in the experiments where high signal genes are being perturbed. Compared to HVG, which is the most common method in the field, 3 out of the 4 GOF variants (PP.ANB, Widst.mean, and Wdist.med) had better overall performance using the crafted experiments while QQ.ANB had better performance than Seurat.vst in the dense gene region. These results also demonstrate that researchers should use any *a priori* knowledge about the biology of important features in their scRNA-seq dataset when choosing a method as different feature selection methods have differing effectiveness for genes with different levels of sparsity.

## Supplementary Material

lqaf023_Supplemental_Files

## Data Availability

In this study, the majority of the data sources was obtained from NCBI Gene Expression Omnibus (GEO; https://www.ncbi.nlm.nih.gov/geo/) (RRID:SCR_005012). The mixture of 3 cell lines dataset is available in GEO: GSE136148 (https://www.ncbi.nlm.nih.gov/geo/query/acc.cgi?acc=GSE136148). The FVB3 mouse mammary gland dataset is available in GEO: GSE136148 (https://www.ncbi.nlm.nih.gov/geo/query/acc.cgi?acc=GSE136148). All the Zheng *et al.* [23] PBMC datasets were downloaded from the R/DuoClustering2018 package [30]. The Zilionis *et al.* human lung dataset [24] was obtained from the R/scRNAseq package (https://www.bioconductor.org/packages/release/data/experiment/html/scRNAseq.html). The crafted datasets based on the mixture of 3 cell lines dataset are available on Github: https://github.com/siyao-liu/CraftedExperiment, and Zenodo: https://zenodo.org/records/13830885. All data used in this study were sourced from publicly accessible repositories that adhered to ethical standards.

## Code availability

GOF is implemented as an open-source R package and is publicly available on Github: https://github.com/siyao-liu/GOF, and Zenodo: https://zenodo.org/records/13830887. Source code including all functions for constructing crafted experiments and benchmarking feature selection methods are publicly available on Github: https://github.com/siyao-liu/CraftedExperiment, and on Zenodo: https://zenodo.org/records/13830885
