# Supplementary material for: Crafted experiments to evaluate feature selection methods for single-cell RNA-seq data
Source: NAR Genom Bioinform. 2025 Mar 19;7(1):lqaf023. doi: 10.1093/nargab/lqaf023 (PMC11920870; doi:10.1093/nargab/lqaf023)
Supplement: lqaf023_Supplemental_Files [file lqaf023_supplemental_files.zip › SupplementalInformation.docx]

**Supplementary information**

**Methods**

###

### Crafted experiments construction

We generated a set of crafted datasets based on the 3-cell line mixture dataset[1] as follows: we created a fourth luminal group by randomly sampling 30% of the luminal cells (N=162) and perturbing a set of genes for these chosen luminal cells. Instead of randomly perturbing genes across the whole genome, we focused on different gene regions based on the relationship between the total count per gene and the proportion of zeros per gene across the luminal cells only. First, we split the genes into three major regions: (1) Sparse (S) - genes with proportion of zeros greater than 0.9 (N=9,223); (2) Medium (M) - genes with proportion of zeros between 0.45 and 0.55 (N=732); (3) Dense (D) - genes with proportion of zeros less than 0.1 (N=613).

From each gene region, we randomly selected 600, 300, 100, and 50 genes to control the strength of the added signal. To allow cross comparisons, these gene sets are nested within each other. For genes in the Sparse region, we added counts modeled by the Poisson distribution with a parameter of 0.5 to the original counts for the selected crafted luminal cells. We feel that additive perturbation is most natural for these sparse genes because these genes tend to have lots of zeros and adding values to zeros is expected to have more impact than a multiplicative perturbation. For the genes in the Medium sparsity region, we applied 3 types of perturbation: adding a count of 2, adding counts modeled by the Poisson distribution with a parameter of 1.5, and adding counts modeled by the Poisson distribution with a parameter taken to be the original count multiplied by 3. For the genes in the Dense region, we applied two kinds of perturbation: adding a count of 10, and adding counts modeled by the Poisson distribution with a parameter taken to be the original count multiplied by 1.5. Thus, in total, we generated a set of 24 crafted datasets on the background of the 3-cell line mixture dataset (**Table 1**).

### GOF methodology

*Average Negative Binomial model*

Multiple recent studies have shown that a simple Poisson model is sufficient for modeling most genes in the scRNA-seq data (especially the UMI count data) [2–6] as opposed to more complex models such as the negative binomial or zero-inflated negative binomial to capture additional variation due to the abundance of zeros in the data[7–9]. Here, we focus on the counts at the individual matrix entry level. In particular, consider a counts matrix $X$ with genes as the rows and cells as the columns. Let $x_{gc}$be the observed count for gene $g=1,2,\ldots, G$ and cell $c=1,2,\ldots,C$*.* We assume that each entry of the original count matrix $X$ is independent and follows a Poisson distribution with its own parameter $\lambda_{gc}:$

$$x_{gc} \sim Poisson (\lambda_{gc})$$

Under this model, the maximum likelihood estimates of $\lambda_{gc}$are not useful, because each is just the single count value $x_{gc}$. This motivates us to put additional structure onto this probability model for improved identifiability of the parameters. In particular, due to technical effects such as sequencing depth or overall mRNA capture efficiency, systematic variation in cell library size (defined as the total counts per cell) is often observed in scRNA-seq data[10]. Such cell-specific variation should be removed from the analysis in order to make biologically meaningful discoveries. One simple and common approach to remove such variations is to apply a global scaling normalization – that is to divide all counts for each cell by a cell-specific size factor[11]. The underlying assumption here is that any cell-specific library size impacts all genes (in terms of $\lambda_{gc})$by a common multiplicative factor. Here, since we are modeling the original counts directly with a probability model, this cell-specific multiplicative factor needs to be incorporated into the corresponding probabilities. Altogether, the reasons above motivate the development of an Average Negative Binomial (ANB) model, which puts additional structure on the Poisson model and recognizes that each cell is driven by a multiplicative effect which is common over genes in that cell.

Specifically, for each gene $g=1,2,\ldots, G,$the ANB distribution models the counts $x_{g1},x_{g2}, \ldots, x_{gC}$ distribution as Negative Binomial (NB), with a mean parameter $\mu_{g}$ and a rate parameter$\phi_{g}$. With this parameterization, the expected value of the NB distribution is $\mu_{g}$ and the variance is$\mu_{g}+ \frac{{\mu_{g}}^{2}}{\phi_{g}}.$ The NB distribution can be considered as a Gamma mixture of Poissons through the random variable $\Lambda_{g}.$ Define the Gamma mixing distribution as $W_{g}$:

$W_{g} \sim Poisson (\Lambda_{g})$, where $\Lambda_{g}\sim Gamma \left( \phi_{g}, \frac{\mu_{g}}{\phi_{g}} \right)$ with shape parameter $\phi_{g}$and scale parameter $\frac{\mu_{g}}{\phi_{g}}$. The expected value of the Gamma distribution is $\mu_{g}$and the variance is $\frac{{\mu_{g}}^{2}}{\phi_{g}}$.

For each gene, the NB parameters $\mu_{g}$ and $\phi_{g}$ can be estimated by maximum likelihood using the fitdist() function in the R/fitdistrplus package[12], resulting in $\hat{\mu_{g}}$and $\hat{\phi_{g}}$, which represent the natural gene variation and over-dispersion for the gene $g$ respectively. In particular, the parameter estimates $\hat{\phi_{g}}$are viewed as driving the common multiplicative variation across cells due to the cell-specific effects such as library size. Because the cell-specific effect is assumed to impact all genes equally by a common multiplicative factor, the individual estimates $\hat{\phi_{1},}\ldots,$ $\hat{\phi_{G}}$ can be pooled into a common estimate $\hat{\phi}$. Instead of fitting the NB with individual parameter estimates, the ANB model fits the pooled estimate $\hat{\phi}$for each gene to incorporate the multiplicative factor that is common across genes, and that is the reason for the name “Average Negative Binomial”. To understand this in a more intuitive way, the parameter estimates $\hat{\phi_{g}}$are considered in terms of the *spread factors* $S_{g}= \frac{1}{\sqrt{\hat{\phi_{g}}}}$ , for $g=1,2,\ldots, G.$ For the estimation of the common spread factor $S$ over genes, the median of the individual estimates of $\hat{S_{g}}$ that are greater than ${10}^{-0.9}$ is taken. The cutoff value ${10}^{-0.9}$ is chosen because there is a dichotomy around ${10}^{-0.9}$ observed in the individual estimates of $\hat{S_{g}}$ in real scRNA-seq datasets. After a careful investigation of the genes with very small estimates of $\hat{S_{g}}$, these very small spread factor estimates seem to be due to some optimization artifacts when obtaining the maximum likelihood estimates for the NB fit. Thus, it is reasonable to remove the spread factor estimates < ${10}^{-0.9}$ from calculating the common spread factor. Define the common spread factor estimate as:

$$\hat{S}={median}_{g\in G} \hat{S_{g}}$$

where $G=\left\{ g=1,\ldots,G \right| \hat{S_{g}}> {10}^{-0.9} \}$.

Altogether, for each gene, the ANB models the counts as the NB distribution with parameters $\mu_{g}$ and $\phi$:

$$W_{g} \sim ANB(\mu_{g}, \phi)$$

where $\mu_{g}$ is the mean parameter and$\phi$ is the common rate parameter ($\phi=\frac{1}{S^{2}}$). The natural variation in genes is accommodated by allowing $\mu$ to be different for each gene, but the common multiplicative effect across genes is captured by the common *spread factor* $S= \frac{1}{\sqrt{\phi}}$.

*Goodness of fit measures*

The P-P plot was constructed as discussed in the “Results” section – Overview of GOF workflow. To generate a version of the Q-Q plot with reasonable performance for small counts, we used the R function developed from Pan et al.’s work[6], which was designed for comparing two samples with a few integers values. Specifically, that Q-Q plot function extrapolates points from integers by using the halves between integers, which returns a Q-Q plot using a more interpretable version of the quantiles.

To compute the Area Between Curves (ABC) in the P-P and Q-Q plots, we first draw vertical lines through each point on the red curve to the golden 45-degree line (**Figure 2** step 2), and then find the total area of the corresponding trapezoids which gives the ABC value. For the Q-Q method, since the axes reflect data quantiles, there is a scaling issue addressed by scaling both axes via dividing them by the sample mean of the theoretical quantiles before calculating the ABC value.

*Discrete 1-Wasserstein distance metric*

First, we introduce notation. Let $n$ be the number of cells. For each gene, let $m$ be the maximum (over cells) count in the gene count distribution, so all of the counts take on one of the values $i=0,1,2,\ldots, m$. For each $i$, let $c_{i}$ denote the number of cells whose count is $i$. We define the empirical probability as $p_{i}=\frac{c_{i}}{n}$. Let$q_{i}$be the theoretical (ANB) probabilities, where $i=0,1,2,\ldots$

Given an empirical distribution $p_{o}, p_{1}, p_{2},\ldots, p_{m}$, and a theoretical distribution $q_{o}, q_{1}, q_{2},\ldots,$ we compute the 1-Wasserstein metric using an interactive algorithm. For convenience, we define $p_{i}=0$ for$i=m+1, m+2,\ldots$ The algorithm starts at step $j=0$ and ${\tilde{p_{0}}=p}_{o}$ and ${\tilde{q_{0}}=q}_{o}$. Then, at step $j=1,2,\ldots,$ the difference between quantile functions (the discrete version of the green and black curves in Figure 3 step 2 bottom right panel) is $D_{j}=\tilde{p}_{j-1}- \tilde{q}_{j-1}$ and the *cost* $C_{j}=|D_{j}|$. Next, we define the updated versions of the probabilities as follows:

If $D_{j} \geq0$, let $\tilde{p}_{j}= p_{j}+ C_{j}$ and let $\tilde{q}_{j}= q_{j}$, otherwise let $\tilde{p}_{j}= p_{j}$and let $\tilde{q}_{j}= q_{j}+ C_{j}$ . Continue the iterations at least until $j=m+1$. After that, continue until $j=k,$ where $k$ is a very large quantile of the ANB distribution, in particular chosen so that $P \left\{ X \leq k \right\} \cong1-{10}^{-5}$, where $X$ has the given ANB distribution.

The 1-Wasserstein distance is the discrete L1-norm defined as $\sum_{j=1}^{nmax} C_{j}$, where $nmax=\max\left( k, m+1 \right).$ Similar to the Q-Q method, due to the scaling issue, we further adjust the 1-Wasserstein distance by the gene average or median. Finally, the top 2000 genes with the largest adjusted distance values are selected for clustering.

**Supplementary Tables**

**Supplementary Table 1.** A list of benchmarked feature selection methods.

| **Method** | **Description** | **Software** |
| --- | --- | --- |
| Seurat.vst | Highly variable genes after variance-stabilized transformation | Seurat V3 |
| Seurat.disp | Highly variable genes based on gene dispersion in respect to gene means | Seurat V3 |
| devianceFS | Feature selection by approximate multinomial deviance | scry |
| HIPPO | Feature selection using zero proportion test | HIPPO |
| PP.ANB | Feature selection based on average negative binomial fit using P-P method | This paper |
| QQ.ANB | Feature selection based on average negative binomial fit using Q-Q method | This paper |
| Wdist.mean | Feature selection based on average negative binomial fit using 1-Wasserstein metric adjusted by gene average | This paper |
| Wdist.med | Feature selection based on average negative binomial fit using 1-Wasserstein metric adjusted by gene median | This paper |

**Supplementary Figures**

**Supplementary Figure 1.** Heatmap of the proportion of the crafted genes selected by each feature selection method in the crafted experiments.

**Supplementary Figure 2.** UMAP of cell populations using the features selected from different feature selection methods in the Zheng4eq dataset. Cell labels are taken from the original publication.

**Supplementary Figure 3.** UMAP of cell populations using the features selected from different feature selection methods in the Zheng4uneq dataset. Cell labels are taken from the original publication.

**Supplementary Figure 4.** UMAP of cell populations using the features selected from different feature selection methods in the Zheng8eq dataset (Note: due to the cut off threshold set in the zero proportion test in HIPPO, there may not be 2000 genes selected by HIPPO). Cell labels are taken from the original publication.

**Supplementary Figure 5.** (A) UMAP of B cells and regulatory T cells using the top 2,000 features selected from different feature selection methods in the Zheng8eq dataset. (B) Scatterplot of gene mean expression across cells on log10 scale and proportion of zeros per gene across cells. Each dot is a gene, and differentially expressed genes (DEG) between the naive T cells and regulatory T cells are highlighted in red triangles. A total of 145 DEGs were identified using the Wilcoxon test implemented in the FindMarkers() function from the R/Seurat package. Grey boxes indicate the Sparse, Medium and Dense regions. The numbers on the right side of each grey box indicate the numbers of DEGs in each region. (C) DiProPerm Z scores and their 95% confidence intervals for each feature selection method from 100 simulations. The mean difference direction between the two groups was computed from the first 30 principal components, which were calculated using the top 2000 selected features from each method. (D) Barplot of the number of genes in the top 2000 genes selected from each feature selection method in Sparse, Medium, and Dense region.

**Supplementary Figure 6.** Comparison of sparsity across datasets. (A) proportion of zeros per gene across cells. (B) proportion of zeros per cell across genes.

**B**

**A**


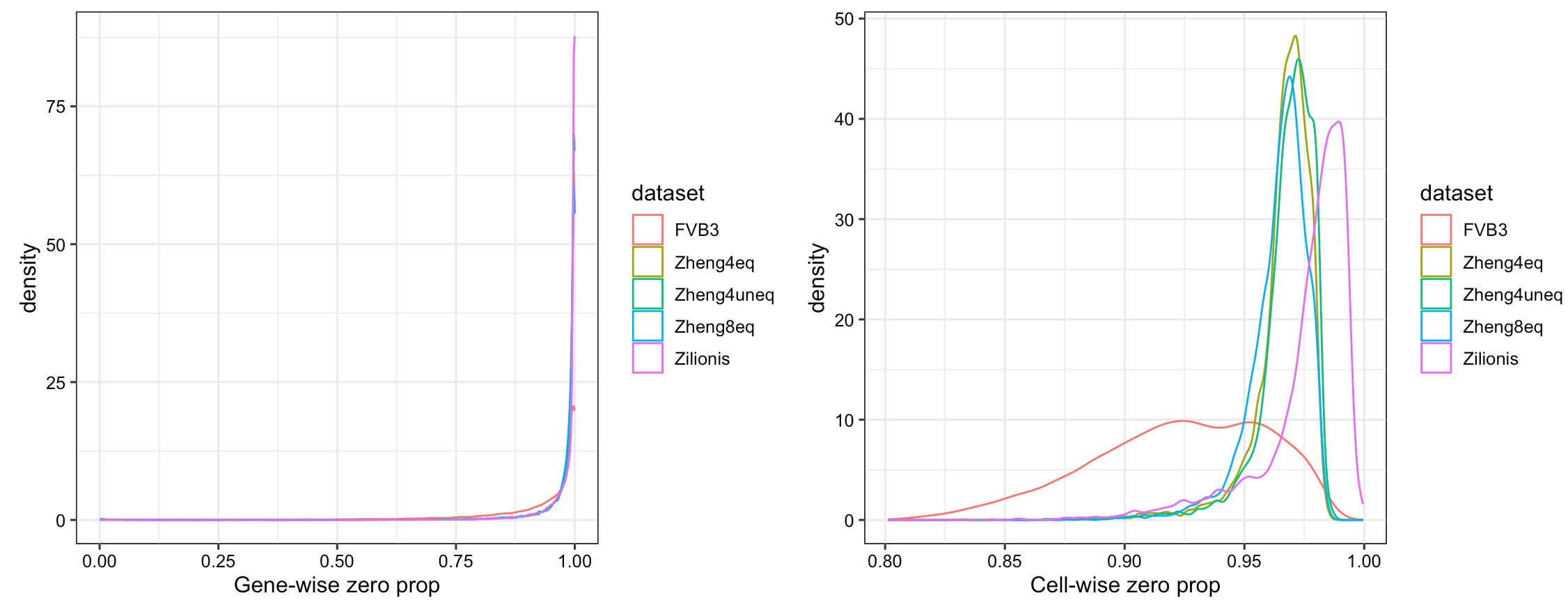


# **References**

1. Dong M, Thennavan A, Urrutia E, Li Y, Perou CM, Zou F, et al. SCDC: bulk gene expression deconvolution by multiple single-cell RNA sequencing references. Briefings in Bioinformatics. 2020. https://doi.org/10.1093/bib/bbz166.

2. Chen W, Li Y, Easton J, Finkelstein D, Wu G, Chen X. UMI-count modeling and differential expression analysis for single-cell RNA sequencing. Genome Biology. 2018. https://doi.org/10.1186/s13059-018-1438-9.

3. Kim TH, Zhou X, Chen M. Demystifying “drop-outs” in single-cell UMI data. Genome Biology. 2020. https://doi.org/10.1186/s13059-020-02096-y.

4. Townes FW, Hicks SC, Aryee MJ, Irizarry RA. Feature selection and dimension reduction for single-cell RNA-Seq based on a multinomial model. Genome Biology. 2019. https://doi.org/10.1186/s13059-019-1861-6.

5. Sarkar A, Stephens M. Separating measurement and expression models clarifies confusion in single-cell RNA sequencing analysis. Nature Genetics. 2021.

6. Pan Y, Landis JT, Moorad R, Wu D, Marron JS, Dittmer DP. The Poisson distribution model fits UMI-based single-cell RNA-sequencing data. BMC Bioinformatics. 2023;24.

7. Pierson E, Yau C. ZIFA: Dimensionality reduction for zero-inflated single-cell gene expression analysis. Genome Biology. 2015. https://doi.org/10.1186/s13059-015-0805-z.

8. Risso D, Perraudeau F, Gribkova S, Dudoit S, Vert JP. A general and flexible method for signal extraction from single-cell RNA-seq data. Nature Communications. 2018. https://doi.org/10.1038/s41467-017-02554-5.

9. Hafemeister C, Satija R. Normalization and variance stabilization of single-cell RNA-seq data using regularized negative binomial regression. Genome Biology. 2019. https://doi.org/10.1186/s13059-019-1874-1.

10. Stegle O, Teichmann SA, Marioni JC. Computational and analytical challenges in single-cell transcriptomics. Nature Reviews Genetics. 2015.

11. Anders S, Huber W. Differential expression analysis for sequence count data. Genome Biology. 2010. https://doi.org/10.1186/gb-2010-11-10-r106.

12. Delignette-Muller ML, Dutang C. fitdistrplus: An R package for fitting distributions. Journal of Statistical Software. 2015. https://doi.org/10.18637/jss.v064.i04.
